# Antioxidant and Antimicrobial Activity of *Citrus sinensis* and *Citrus aurantium* Essential Oil Against Foodborne Bacterial Pathogens

**DOI:** 10.3390/foods15213867

**Published:** 2026-10-29

**Authors:** Juan Gaibor-Chávez, Orlando Meneses-Quelal, Franz Verdezoto-Mendoza, Angélica Tigre-León, Favian Bayas-Morejón, Sthefany Gaibor-Cruz

**Affiliations:** 1 Carrera de Agroindustria, Facultad de Ciencias Agropecuarias, Recursos Naturales y del Ambiente, Universidad Estatal de Bolívar (UEB), Guaranda 020150, Ecuadorfbayas@ueb.edu.ec (F.B.-M.);; 2 Carrera de Alimentos, Facultad de Industrias Agropecuarias y Ciencias Ambientales, Universidad Politécnica Estatal del Carchi (UPEC), Tulcán 40101, Ecuador

**Keywords:** *Citrus sinensis*, *Citrus aurantium*, essential oil, antioxidant activity, antimicrobial activity, agro-industrial waste valorization

## Abstract

Orange peel is a citrus-processing by-product and a promising source of volatile compounds for value-added applications. This study compared essential oils obtained by steam distillation from Valencia orange (*Citrus sinensis*) and sour orange (*Citrus aurantium*) peel under a common experimental framework. Antioxidant capacity was assessed independently by ABTS and FRAP, whereas antimicrobial activity was screened by disk diffusion against *Escherichia coli*, *Shigella* spp., *Staphylococcus aureus*, and *Bacillus cereus*. Both citrus materials yielded 1.25 mL essential oil/100 g peel. In the prepared oil batches, Valencia orange essential oil showed the higher antioxidant response in both assays, reaching 438.08 ± 6.50 µmol TE/L under ABTS and 187.21 ± 13.11 µmol TE/L under FRAP, approximately 2.80- and 2.62-fold the corresponding sour orange values. Sour orange essential oil produced inhibition zones against all four bacteria (16.0–29.5 mm), whereas no inhibition zone was detected for Valencia orange oil under the same screening conditions. Under this defined workflow, the two prepared oils showed contrasting functional response profiles, providing a quantitative basis for targeted compositional and application-oriented follow-up.

## 1. Introduction

Citrus processing generates a substantial stream of peel, pulp, seeds, and residual membranes that can be redirected from low-value disposal toward the recovery of functional compounds. Citrus peel is especially attractive in this context because it contains volatile terpenes, pectin, flavonoids, pigments, and other constituents that can support integrated valorization strategies. Recent life-cycle, packaging, biorefinery, and microbial-valorization studies have therefore positioned citrus residues as a relevant feedstock for circular bioeconomy approaches in the agri-food sector [1,2,3,4]. Recovering essential oils from the flavedo is one of the most direct routes because it combines the use of a renewable processing residue with the production of a concentrated volatile fraction that can be evaluated for technological and biological functionality.

The biological performance of citrus peel essential oils is strongly dependent on botanical identity and processing history. Species, cultivar, maturity stage, environmental conditions, plant organ, storage, pretreatment, and extraction method can modify both the qualitative and quantitative volatile profiles. Reviews and experimental studies on *Citrus sinensis* and *Citrus aurantium* have repeatedly shown that limonene is frequently prominent, while the proportions of oxygenated monoterpenes, pinenes, linalool, γ-terpinene, and other minor constituents can vary substantially among materials and extraction conditions [5,6,7,8,9]. This compositional variability is scientifically important because essential oil activity reflects the combined behavior of the volatile mixture rather than the presence of a single constituent in isolation.

Valencia sweet orange (*C. sinensis*) and sour orange (*C. aurantium*) are relevant citrus materials in Ecuador and represent a useful pair for comparative functional assessment. Antibacterial, antifungal, antioxidant, and antibiofilm responses have been reported for oils or extracts from both species, but the magnitude and direction of these responses differ across studies according to the plant material, extraction procedure, assay system, dose, and target microorganism [10,11,12,13,14,15]. A side-by-side experiment in which the two oils are generated and assessed under the same laboratory workflow can therefore reveal whether the two citrus materials express similar or differentiated functional patterns when methodological variability is reduced within the study.

Antioxidant activity is not a single chemical property and is commonly evaluated through complementary assays that probe different reaction principles. The ABTS radical-cation decolorization assay measures the capacity of sample constituents to quench the preformed ABTS radical cation and has broad applicability to hydrophilic and lipophilic antioxidant systems [16]. The FRAP assay, in contrast, estimates ferric-to-ferrous reducing capacity under acidic conditions through formation of the colored Fe^2+^–TPTZ complex [17]. Using both assays as separate endpoints is therefore informative for essential oils because agreement in the direction of the response across two mechanistically distinct analytical systems provides a broader functional description than either assay alone. In the present study, ABTS and FRAP were consequently interpreted independently rather than as interchangeable levels of one statistical factor.

The antimicrobial component of this study focused on *Escherichia coli*, *Shigella* spp., *Staphylococcus aureus*, and *Bacillus cereus*. These organisms provide a diverse screening panel that includes Gram-negative and Gram-positive bacteria of relevance to food microbiology. Disk diffusion remains a practical screening approach for visualizing inhibition around an applied antimicrobial material, although the magnitude and morphology of the zone are influenced by agar conditions and diffusion behavior [18,19]. The inclusion of both Gram classes is useful because bacterial-envelope architecture can influence access of lipophilic essential oil constituents to the cytoplasmic membrane. Independent studies with *Shigella* and *Bacillus* models have likewise shown that plant-derived antimicrobials can generate measurable inhibition through membrane-associated and other cellular effects [20,21,22,23].

Mechanistic research on citrus volatiles provides additional context for interpreting functional differences between oils. Limonene has been studied extensively and can perturb bacterial membrane integrity, modify permeability, and promote leakage of intracellular material in model systems involving *E. coli* and *S. aureus* [24,25,26]. Formulation work has further shown that the antimicrobial behavior of d-limonene can be maintained in stabilized dispersed systems [27]. These findings are relevant as a literature framework for citrus oils, while the functional response of any specific oil remains a property of its complete sample-specific composition. For that reason, the present work centers on the measured antioxidant and inhibition-zone responses of the prepared oils and uses constituent-level evidence only to contextualize plausible directions for subsequent composition–activity analysis.

Direct comparisons between Valencia orange and sour orange essential oils obtained under a common extraction protocol and tested across both antioxidant and antimicrobial endpoints remain comparatively scarce. The present study therefore aimed to characterize the functional differentiation of steam-distilled essential oils from Valencia orange and sour orange peel by (i) determining volumetric extraction yield, (ii) evaluating antioxidant response independently by ABTS and FRAP, and (iii) describing in vitro inhibition-zone responses against *E. coli*, *Shigella* spp., *S. aureus*, and *B. cereus*. The comparison is interpreted at the level of the prepared oil batches and the analytical responses generated under the shared workflow.

## 2. Materials and Methods

### 2.1. Study Site and Plant Material

This study was conducted in the General Laboratory of the Faculty of Agricultural Sciences, Natural Resources and the Environment and the Research and Outreach Laboratory of the Universidad Estatal de Bolívar, Guaranda canton, Bolívar province, Ecuador (zone 17M, east 722773, north 9821524.1). The plant material was identified in the experimental record as Valencia orange (*C. sinensis*) and sour orange (*C. aurantium*) obtained from Caluma canton, Bolívar province. Approximately 40 fruits were selected for a sound external condition and comparable apparent ripeness so that visibly damaged, deteriorated, or markedly heterogeneous material was excluded from processing. The fruits were washed under running water, manually peeled, and the flavedo was chopped immediately before pretreatment. A mass of 400 g of prepared peel was used for each recorded extraction. The same selection and preparation sequence was applied to both citrus materials to maintain a common handling framework before essential oil recovery. For manuscript reporting, the citrus materials are retained under the taxonomic names documented in the experimental record, without extending identification beyond the recorded source information.

### 2.2. Essential Oil Extraction

Essential oil was recovered by steam distillation, a conventional approach for isolating the volatile fraction of aromatic plant matrices and citrus peel. Distillation-based extraction is widely used because water and steam can transfer volatile compounds from plant tissues without requiring an organic extraction solvent [28,29]. For each recorded extraction, 400 g of chopped peel was combined with 500 mL of distilled water containing 0.7% (3.5 g) sodium bicarbonate and left to macerate for 8–12 h at room temperature. The sodium-bicarbonate pretreatment was intended to promote hydration and partial disruption of pectic structures in the flavedo, thereby facilitating release from the oil glands. A comparable citrus-peel pretreatment and operational rationale has been reported in the related biomass-valorization work of Quilo Catucuamba et al. [30], and pre-extraction hydration/maceration is consistent with broader green-extraction and citrus-peel pretreatment approaches [28,31]. After maceration, the material was ground, transferred to the distillation apparatus with an additional 15% (60 mL) of distilled water, and heated to boiling without exceeding 100 °C. Condensate was collected for 65 min and transferred to a stoppered separatory funnel for 24 h so that the essential oil and aqueous phases could separate by density. The oil phase was collected into labeled amber-glass vials and kept protected from light in the laboratory’s cool-storage environment until analysis. The same extraction sequence was applied to both citrus materials. Volumetric extraction yield (R, mL/100 g) was calculated as shown in Equation (1), consistent with volume-to-mass reporting used for essential oil recovery [30]:

*R* = (*V*_1_/*M*_2_) × 100, (1)

where *V*_1_ is the volume of essential oil recovered (mL) and *M*_2_ is the initial mass of processed peel (g). Yield is expressed on a volume/mass basis because the recovered oil was quantified volumetrically.

### 2.3. Functional Characterization Framework

The experimental program was structured as a comparative functional characterization of the two prepared citrus essential oils. Antioxidant response was assessed through two independent chemical assays, ABTS and FRAP, whereas antimicrobial response was assessed through inhibition-zone measurements against four bacterial targets. This structure allows each analytical endpoint to retain its own chemical or microbiological meaning while still supporting a direct within-study comparison between the two citrus materials. The literature on citrus volatile constituents is used in the Discussion to place the functional patterns in a mechanistic context; the measured endpoints themselves are the basis of the experimental comparison.

Figure 1 illustrates the extraction workflow and representative experimental stages using process-only images, from raw-material preparation to phase separation and antioxidant-assay calibration.

### 2.4. Analytical Replication and Data Structure

For antioxidant characterization, three analytical determinations were performed for each oil–assay combination, giving 12 measurements in total across Valencia orange, sour orange, ABTS, and FRAP. These repeated determinations describe within-batch analytical consistency under the corresponding assay conditions. The raw observations are reported together with mean and standard deviation so that the measurement structure remains fully visible in the Results.

For antimicrobial characterization, two analytical determinations were recorded for each oil–microorganism combination, giving 16 inhibition-zone measurements across the two oils and four bacterial targets. The two observations for each condition are reported individually and summarized descriptively. The experimental unit for each citrus material was the prepared oil batch; consequently, *n* = 2–3 denotes analytical repetition, and the comparisons are framed as within-study contrasts between the two prepared oil batches rather than as population-level species estimates (Table 1).

### 2.5. Determination of Antioxidant Activity

Antioxidant activity was first determined with the ABTS radical-cation decolorization principle described by Re et al. [16], using the reagent volumes and incubation conditions recorded in the laboratory protocol. The ABTS cation radical was generated by mixing 5 mL of ABTS solution (7 mM) with 88 µL of potassium persulfate (140 mM) and allowing the mixture to react in the dark for 12 h. The resulting solution was diluted with acetic acid–sodium acetate buffer (pH 5) until an absorbance of 0.7 was reached at 734 nm. A 190 µL aliquot of working solution was mixed directly with 10 µL of essential oil, allowed to react for 5 min at room temperature, and measured at 734 nm with a NanoDrop spectrophotometer (Thermo Scientific, Waltham, MA, USA). Trolox standards covering 0–200 µmol/L were used for calibration, and the antioxidant response was expressed as µmol Trolox equivalents (TE) per liter of sample. All samples were handled using the same sequence, reaction time, and instrumental reading conditions.

Ferric reducing antioxidant power was assessed using the FRAP principle originally described by Benzie and Strain [17], adapted to the reagent volumes recorded for the present experiment. The working reagent was prepared by combining 2.5 mL of sodium acetate buffer, 2.5 mL of FeCl_3_ solution, and 2.5 mL of TPTZ solution and was incubated for 30 min at 37 °C protected from light. Reaction tubes contained 30 µL of distilled water, 30 µL of essential oil, and 900 µL of FRAP reagent. For the reagent blank, the essential oil aliquot was replaced with 30 µL of ethanol. After a further 30 min at 37 °C, absorbance was measured at 593 nm. Trolox standards were prepared in 96% ethanol, and the response was expressed as µmol TE/L. ABTS and FRAP were treated as separate analytical endpoints because they quantify different antioxidant reaction mechanisms [16,17]. The recorded direct oil–reagent procedure was applied identically to both prepared oils; the resulting values are therefore interpreted as assay-specific comparative responses under this fixed preparation sequence.

### 2.6. Determination of Antimicrobial Activity

Institutional bacterial isolates recorded as *E. coli*, *S. aureus*, *Shigella* spp., and *B. cereus* were obtained from the microorganism bank associated with the General Laboratory of the Faculty of Agricultural Sciences, Natural Resources and the Environment and the Research and Outreach Laboratory of the Universidad Estatal de Bolívar, where they had been previously characterized. The bacteria were reactivated on selective media appropriate to the recorded laboratory workflow: MacConkey agar for *E. coli*, mannitol salt agar for *S. aureus*, Salmonella-Shigella agar for *Shigella* spp., and chromogenic Bacillus agar for *B. cereus*. Media preparation, sterilization at 121 °C for 15 min, plate handling, and incubation procedures followed the laboratory record and the general microbiological examination guidance of ISO 7218 [32]. Cultures were incubated at 37 °C for 24 h before use. Antimicrobial response was then screened by disk diffusion, a method in which the diameter of the clear growth-inhibition zone surrounding an impregnated disk provides a direct comparative endpoint; the importance of standardized agar and zone-reading conditions in disk diffusion has been documented in methodological studies [18,19]. Six-millimeter paper disks received the nominal essential oil amounts recorded in the experimental protocol (5, 10, and 15 µg per disk), and a blank disk without oil was included as the negative control. Bacterial suspensions were adjusted to 0.5 McFarland before inoculation. After incubation, the clear-zone diameter was measured in millimeters. The same oil-testing sequence was used for the two citrus materials and all four bacterial targets. The manuscript reports the isolates at the bank-identification level preserved in the experimental record and does not assign a strain-level designation beyond that documented identity. Accordingly, inhibition-zone diameter is used as a screening endpoint; numerical zone differences are not interpreted as direct measures of intrinsic antimicrobial potency because diffusion and physicochemical behavior can also influence zone formation.

### 2.7. Statistical Analysis

Statistical analysis was structured according to the hierarchy of the recorded measurements and was conducted in R version 4.6.1 (R Foundation for Statistical Computing, Vienna, Austria) using RStudio 2026.08.0 ‘Yellow Yarrow’ (Posit Software, PBC, Boston, MA, USA). ABTS and FRAP were analyzed as separate antioxidant endpoints because they quantify different reaction mechanisms. For each oil–assay combination, the three analytical determinations were summarized by the arithmetic mean, standard deviation (SD), relative standard deviation (RSD = 100 × SD/mean), observed range, absolute between-oil difference, and Valencia-to-sour orange fold ratio. These descriptors quantify the magnitude of the observed contrast and analytical repeatability within each prepared oil batch without treating repeated measurements as independent biological or extraction units. For the antimicrobial screening, the two inhibition-zone observations for every oil–microorganism condition were retained individually and summarized by mean ± SD; zero values were preserved as observed, without transformation or continuity correction. Factorial ANOVA, normality and homogeneity tests, post hoc multiple comparisons, permutation *p*-values, and null-hypothesis significance terminology were not applied. All ratios and absolute contrasts were calculated from the unrounded group means, and tabulated summaries were checked against the individual observations before interpretation. This strategy preserves the complete measurement pattern, reports analytical dispersion explicitly, and keeps inference aligned with the batch-level experimental structure.

## 3. Results

### 3.1. Essential Oil Yield

Under the recorded extraction conditions, 400 g of peel yielded 5 mL of essential oil for both Valencia orange and sour orange, corresponding to an observed volumetric yield of 1.25 mL/100 g for each material. During the recorded distillation run, condensation began at approximately 23 °C for Valencia orange peel and 10 °C for sour orange peel and subsequently reached a common 60–70 °C range during the 65 min condensate-collection period. The equal final recovered volume indicates that, under the shared protocol, the two peel materials delivered the same volumetric oil yield even though the onset of condensation differed. These process observations provide the operational context for comparing the subsequent antioxidant and antimicrobial responses of the recovered oils.

### 3.2. Antioxidant Activity

Table 2 reports the individual analytical determinations for each oil–assay condition together with the descriptive summaries, and Figure 2 displays the corresponding means. The two antioxidant assays are treated as independent endpoints in accordance with their distinct reaction principles.

In ABTS, Valencia orange essential oil produced 431.580, 438.080, and 444.582 µmol TE/L, yielding a mean of 438.08 ± 6.50 µmol TE/L. Sour orange essential oil produced 158.514, 152.012, and 158.514 µmol TE/L, with a mean of 156.35 ± 3.75 µmol TE/L. The observed absolute difference between the two oils was therefore 281.73 µmol TE/L, and the Valencia-to-sour orange ratio was 2.80.

The same direction of response was observed in FRAP. Valencia orange essential oil produced 200.31, 187.21, and 174.10 µmol TE/L, corresponding to 187.21 ± 13.11 µmol TE/L, whereas sour orange essential oil produced 69.27, 69.27, and 75.82 µmol TE/L, corresponding to 71.45 ± 3.78 µmol TE/L. The observed difference was 115.76 µmol TE/L and the Valencia-to-sour orange ratio was 2.62. Thus, although ABTS and FRAP represent different antioxidant mechanisms and are not compared statistically with one another, both independently showed the same between-oil ordering: Valencia orange essential oil had the higher antioxidant response under the recorded assay conditions.

Analytical dispersion was low to moderate across the four oil–assay conditions: RSD values were 1.48% for Valencia orange in ABTS, 2.40% for sour orange in ABTS, 7.00% for Valencia orange in FRAP, and 5.29% for sour orange in FRAP. Reporting RSD together with the individual values and SD provides an explicit measure of within-sample analytical repeatability while preserving the between-oil effect magnitude as the main comparative result.

### 3.3. Antimicrobial Activity

Table 3 presents the individual inhibition-zone observations and descriptive summaries for the antimicrobial screening, and Figure 3 displays the corresponding means. Reporting both raw values and descriptive summaries makes the response pattern for each oil–microorganism condition directly traceable; zone diameters are interpreted as screening responses rather than as direct estimates of intrinsic antimicrobial potency.

Sour orange essential oil produced measurable inhibition zones against all four bacteria, whereas Valencia orange essential oil produced no detectable inhibition under the recorded conditions. The two sour orange observations were 20 and 19 mm for *E. coli*, 16 and 16 mm for *Shigella* spp., 30 and 29 mm for *S. aureus*, and 30 and 29 mm for *B. cereus*. The corresponding Valencia orange observations were 0 and 0 mm for each bacterium. The observed sour orange inhibition range therefore extended from 16.0 to 29.5 mm when expressed as condition means.

Within the sour orange dataset, the largest observed mean inhibition zones were 29.5 mm for both *S. aureus* and *B. cereus*, followed by 19.5 mm for *E. coli* and 16.0 mm for *Shigella* spp. These values describe the observed screening magnitudes under the recorded disk-diffusion conditions. Because oil diffusion and physicochemical behavior contribute to zone formation, the numerical ordering is not used as a direct potency ranking.

## 4. Discussion

The present study revealed differentiated functional responses from the two prepared citrus-peel oil batches processed under a shared extraction workflow: equivalent observed volumetric oil recovery, a higher antioxidant response for the Valencia orange batch in both antioxidant assays, and measurable antibacterial inhibition only for the sour orange batch under the disk-diffusion conditions used. Evaluating the materials through the same experimental sequence reduces within-study methodological variation and supports a direct comparison of the measured responses. The interpretation throughout this section remains at the level of the prepared batches and the specified analytical workflow.

The observed yield of 1.25 mL/100 g for both oils falls within the broad process-dependent variability reported for citrus and other aromatic plant matrices. Essential oil recovery can change with species, gland density, particle size, hydration, maceration, temperature, distillation configuration, extraction time, and the way yield is expressed [28,29,30,31]. The related study by Quilo Catucuamba et al. [30], conducted on citrus biomass from the same Ecuadorian province, likewise demonstrated that variety and maceration conditions can generate substantial differences in essential oil recovery. In the present comparison, however, the two materials converged on the same final volumetric yield. This is technologically relevant because it shows that a similar recovered oil volume does not imply similar functional behavior: subsequent antioxidant and antimicrobial testing separated the two oils clearly despite equivalent volumetric recovery.

The antioxidant results showed the same between-batch ordering in both assays. Valencia orange essential oil exceeded sour orange essential oil by 281.73 µmol TE/L in ABTS and 115.76 µmol TE/L in FRAP, corresponding to observed ratios of 2.80 and 2.62, respectively. Comparable species- and process-dependent variation in antioxidant response has been reported for citrus peel extracts and essential oils [33,34,35,36]. Alarcon Mite et al. [33] and Jiménez Jiménez et al. [34] documented marked differences among citrus materials, while Lai et al. [35] showed that postharvest handling can modify orange-peel antioxidant characteristics. De Aquino et al. [36] further reported antioxidant effects of *C. sinensis* peel essential oil in a biological model. These studies provide literature context for the variability expected across citrus materials and processing histories without assigning the present batch-level contrast to a single constituent or species-wide property.

ABTS and FRAP contribute complementary information because ABTS reflects radical-cation quenching, whereas FRAP reflects reducing capacity toward a ferric complex [16,17]. Their numerical scales are therefore not treated as equivalent measures of one underlying quantity. The direct oil–reagent procedure was applied identically to both prepared oils, so the reported values are interpreted as method-specific comparative responses under a fixed assay configuration. Within that defined framework, both independent assays produced the same between-oil ordering, providing a cross-assay observation without assuming equivalence between the two analytical principles.

The descriptive statistics separate the magnitude of the within-study contrasts from analytical dispersion. Across the antioxidant measurements, the fold contrasts between the two prepared batches (2.80 in ABTS and 2.62 in FRAP) were larger than the within-condition variation summarized by the RSD values. In the antimicrobial screening, paired readings within each sour orange condition differed by no more than 1 mm, while the Valencia orange readings were 0 mm for all four bacterial targets. These analytical repetitions characterize measurement consistency within the prepared batches and are not used to estimate variation among independent extractions. Reporting the complete raw observations together with SD and RSD keeps the quantitative interpretation aligned with the recorded data structure.

Citrus peel as a whole also contains abundant non-volatile phenolics and flavanones, and hesperidin is particularly well documented in sweet orange peel [37,38]. Steam-distilled essential oil, however, represents the volatile fraction of that matrix, so peel-level flavonoid abundance and essential oil antioxidant response should be considered related but distinct aspects of citrus valorization. This distinction is useful for process design. A biorefinery strategy can recover volatile oil as one product stream while directing the residual peel toward pectin, hesperidin, fiber, or other non-volatile fractions [3,37]. The measured antioxidant response for the Valencia orange batch therefore adds a functional property to one fraction of a broader citrus-peel valorization cascade rather than requiring a single compound to account for the entire response.

The antimicrobial screening produced a clear within-study contrast. The sour orange batch generated measurable zones against all four tested bacteria, whereas the Valencia orange batch produced no detectable zone in either analytical determination. Published studies confirm that both *C. aurantium* and *C. sinensis* can exhibit antimicrobial activity, while also documenting wide variation associated with cultivar, extraction route, volatile profile, applied amount, diffusion behavior, and bacterial strain [10,11,12,13,14,15]. The present observations are therefore interpreted as batch-specific screening responses under the common assay protocol rather than as species-level performance estimates.

Within the sour orange screening, *S. aureus* and *B. cereus* produced observed mean zones of 29.5 mm, followed by *E. coli* (19.5 mm) and *Shigella* spp. (16.0 mm). This numerical pattern is reported descriptively and is not interpreted as a direct hierarchy of antimicrobial potency, because disk-diffusion zones reflect both biological inhibition and the diffusion behavior of the applied material [18,19]. Bacterial-envelope architecture nevertheless provides relevant mechanistic context: Gram-negative bacteria possess an outer membrane containing lipopolysaccharide that can restrict the penetration of hydrophobic molecules, whereas Gram-positive cells lack this outer membrane and expose the peptidoglycan-associated surface more directly to lipophilic compounds. Essential oil and plant-derived antimicrobial studies with *Shigella* and *Bacillus* models have reported membrane perturbation, growth inhibition, and related cellular responses [20,21,22,23].

Limonene provides one of the best-characterized mechanistic models for interpreting citrus-oil antimicrobial action. Independent experiments have shown that limonene can disrupt membrane and cell-wall integrity, increase permeability, and promote leakage of intracellular macromolecules in *E. coli* and *S. aureus* [24,25,26]. Nanoemulsion research additionally shows that the formulation can influence the stability and effective delivery of limonene in aqueous environments [27]. These studies provide a mechanistic framework for citrus essential oils, but they are not used to assign limonene content or constituent-specific effects to the prepared oils evaluated here.

Table 4 situates the observed yield and bioactivity values alongside selected previous studies on *C. sinensis* and *C. aurantium*. The comparison is intended as qualitative scientific context because the plant organ, extraction method, dose, microbial strain, and assay configuration differ among studies.

Table 4 places the present values beside selected published studies and illustrates how strongly the reported antimicrobial magnitude depends on the experimental context. Anwar et al. [10] reported measurable inhibition for *C. sinensis* peel oil obtained by hydrodistillation, while Abud Espinoza et al. [39] observed activity from seed-derived oils extracted with ethanol. Ellouze et al. [15] reported substantial *E. coli* inhibition for *C. aurantium* branch essential oil and further evaluated MIC. These comparisons do not imply numerical equivalence because the plant organ, extraction method, applied dose, microbial strain, and assay configuration differ; they document the broad range of antimicrobial responses reported across citrus materials and experimental systems.

### Composition–Activity Context

Published compositional studies provide quantitative context for the functional profiles while demonstrating how strongly the essential oil composition can depend on extraction conditions. In *C. aurantium* peel oil, Ashmawy et al. [6] identified limonene as 98.86% after steam distillation, 98.68% after hydrodistillation, and 99.23% after microwave-assisted hydrodistillation; the hydrodistilled fraction also contained linalool (0.12%) and α-terpineol acetate (0.10%), constituents discussed by those authors in relation to differences among antioxidant and enzyme-inhibition responses. For *C. sinensis* peel oil, Hacib et al. [40] reported limonene at 78.192% after hydrodistillation and 58.6% after microwave extraction, together with method-dependent antioxidant and antibacterial responses. A *C. sinensis* cv. New Hall–*C. aurantium* pericarp oil was reported to contain limonene (76.25%), β-pinene (4.14%), linalool (1.77%), α-terpineol (1.29%), myrcene (1.12%), valencene (1.15%), and α-pinene (1.07%), alongside antimicrobial activity [11]. These published percentages are included as composition–activity context and are not assigned to the oils analyzed in the present study; constituent-level attribution of the present responses is reserved for direct compositional profiling of the corresponding oil batches.

From an application perspective, the observed within-study responses suggest distinct priorities for subsequent validation. The Valencia orange batch provides a quantitative starting point for follow-up antioxidant evaluation, whereas the sour orange batch provides a starting point for follow-up antimicrobial screening. Citrus essential oils and peel-derived constituents have been incorporated into films, coatings, nanoemulsions, and preservation-oriented systems [41,42,43,44,45], and compositional stability during storage and processing is an active area of essential oil research [46]. The present results can therefore be used to define the next composition, formulation, stability, and food-matrix experiments without treating the two citrus materials as functionally interchangeable.

This differentiated valorization strategy is consistent with circular-bioeconomy principles. Instead of treating citrus peel as a uniform processing residue, experimentally observed functionality can guide the design of higher-value follow-up pathways. The two oil batches and the remaining non-volatile peel matrix can be considered complementary streams within an integrated processing concept, linking waste reduction with functional-ingredient research and subsequent validation in food-relevant systems.

## 5. Conclusions

Under the shared steam-distillation conditions employed, the prepared Valencia orange and sour orange peel batches each yielded 1.25 mL essential oil/100 g peel. Within the analytical dataset, Valencia orange essential oil showed the higher antioxidant response in both independent assays, reaching 438.08 ± 6.50 µmol TE/L in ABTS and 187.21 ± 13.11 µmol TE/L in FRAP, compared with 156.35 ± 3.75 and 71.45 ± 3.78 µmol TE/L, respectively, for sour orange. Sour orange essential oil produced measurable disk-diffusion zones against all four bacteria, with means of 19.5 mm for *E. coli*, 16.0 mm for *Shigella* spp., and 29.5 mm for both *S. aureus* and *B. cereus*, whereas no inhibition zone was detected for the Valencia orange batch under the same screening conditions. These findings define two differentiated response profiles under the specified workflow. Rather than implying species-wide performance, the results support prioritizing the Valencia-derived oil for follow-up antioxidant studies and the sour orange-derived oil for follow-up antimicrobial studies. The dataset provides a defined experimental basis for direct compositional profiling, replication across independent extraction batches, formulation studies, and validation in food-relevant systems.

## Figures and Tables

**Figure 1:**
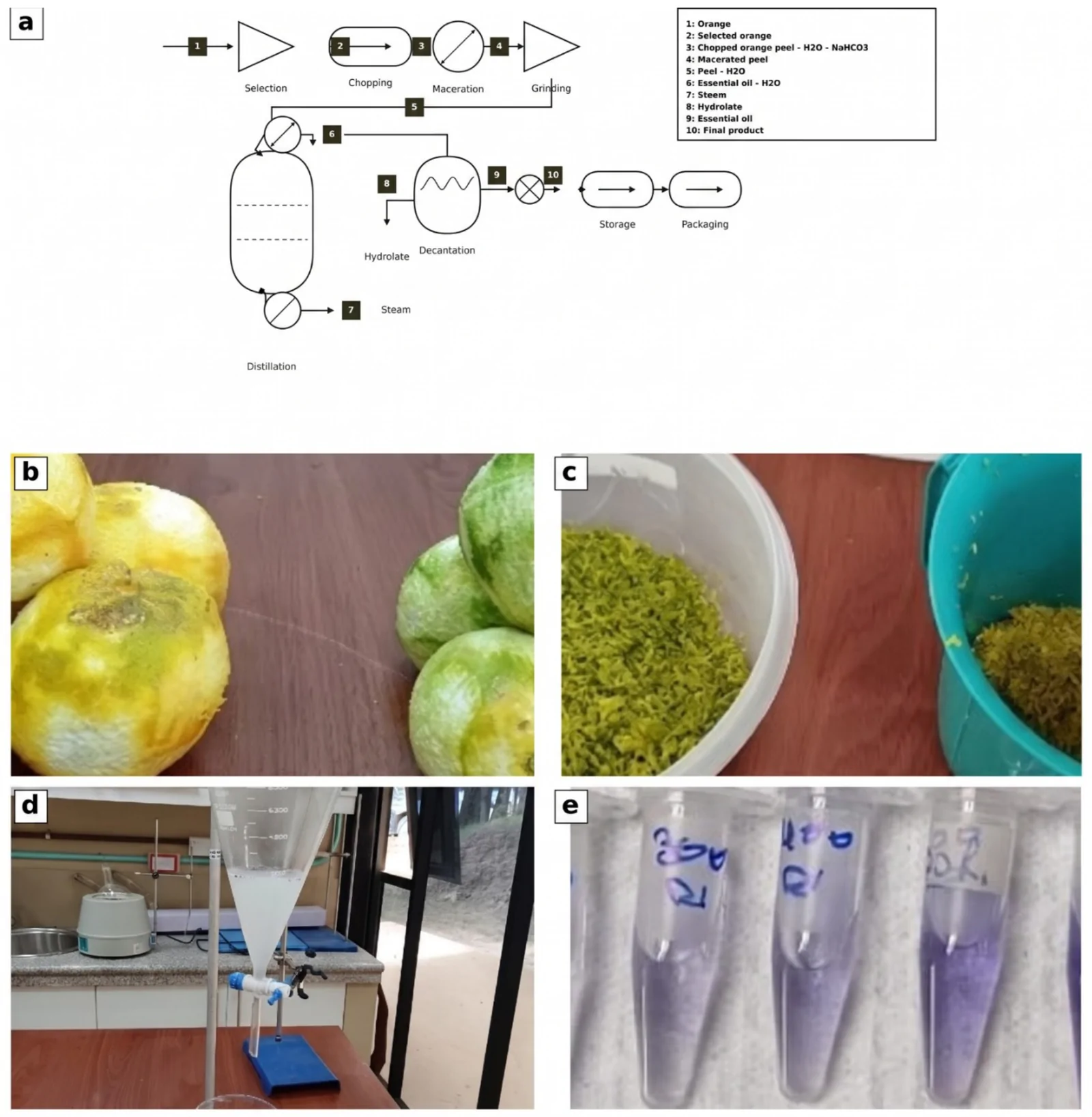
Flow diagram of the extraction process and representative experimental stages. (**a**) Flow diagram of the essential oil extraction process. (**b**) Valencia orange peel (left) and sour orange peel (right) after manual peeling. (**c**) Chopped and ground peel prior to maceration. (**d**) Separation of the essential oil and aqueous phase (hydrolate) using a separating funnel. (**e**) Calibration-curve vials used for antioxidant measurement.

**Figure 2:**
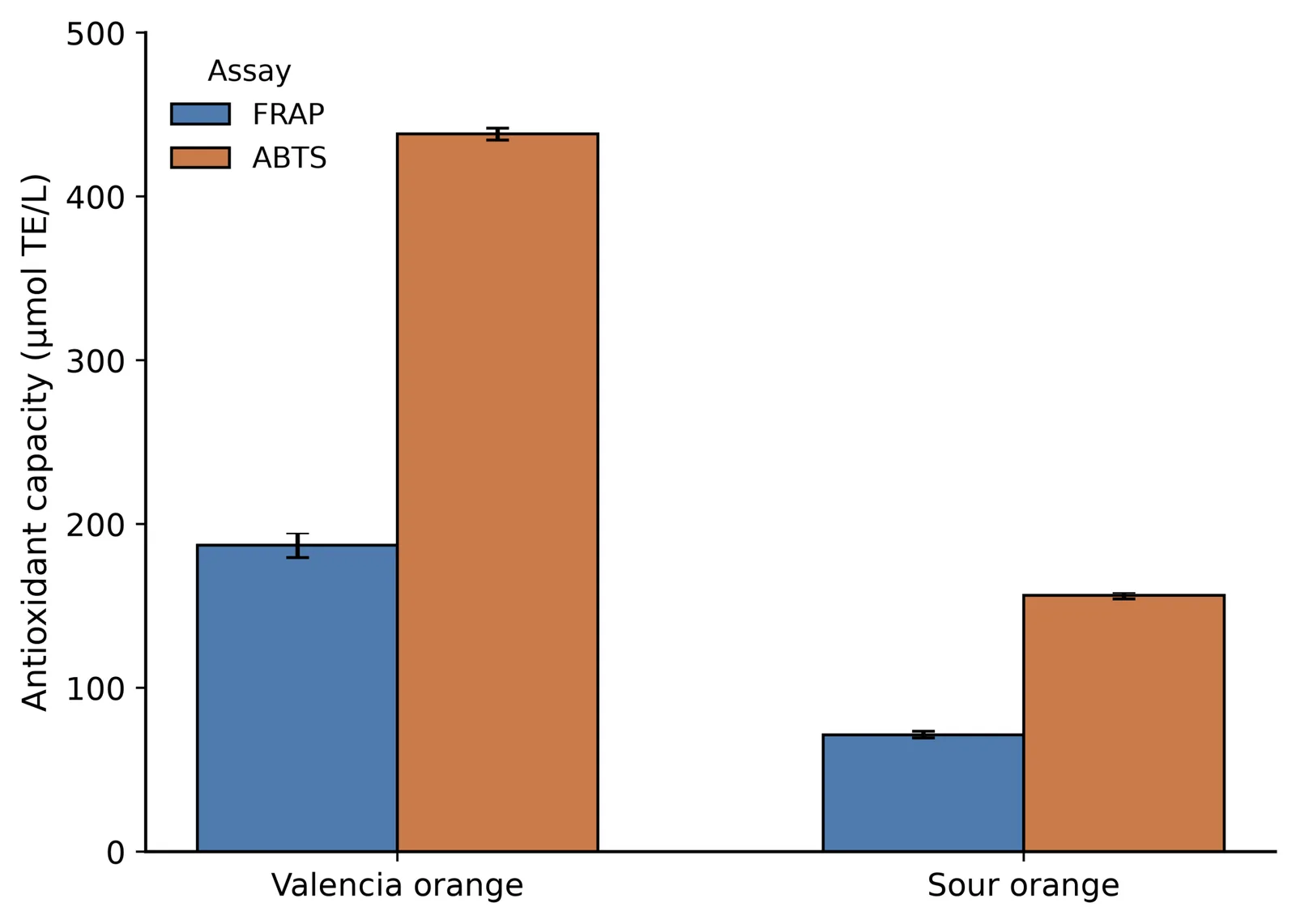
Antioxidant capacity of Valencia orange and sour orange essential oil determined by the FRAP and ABTS assays. Bars represent the mean ± standard error of three analytical determinations per oil–assay condition. Error bars summarize within-sample analytical dispersion. TE = Trolox equivalents.

**Figure 3:**
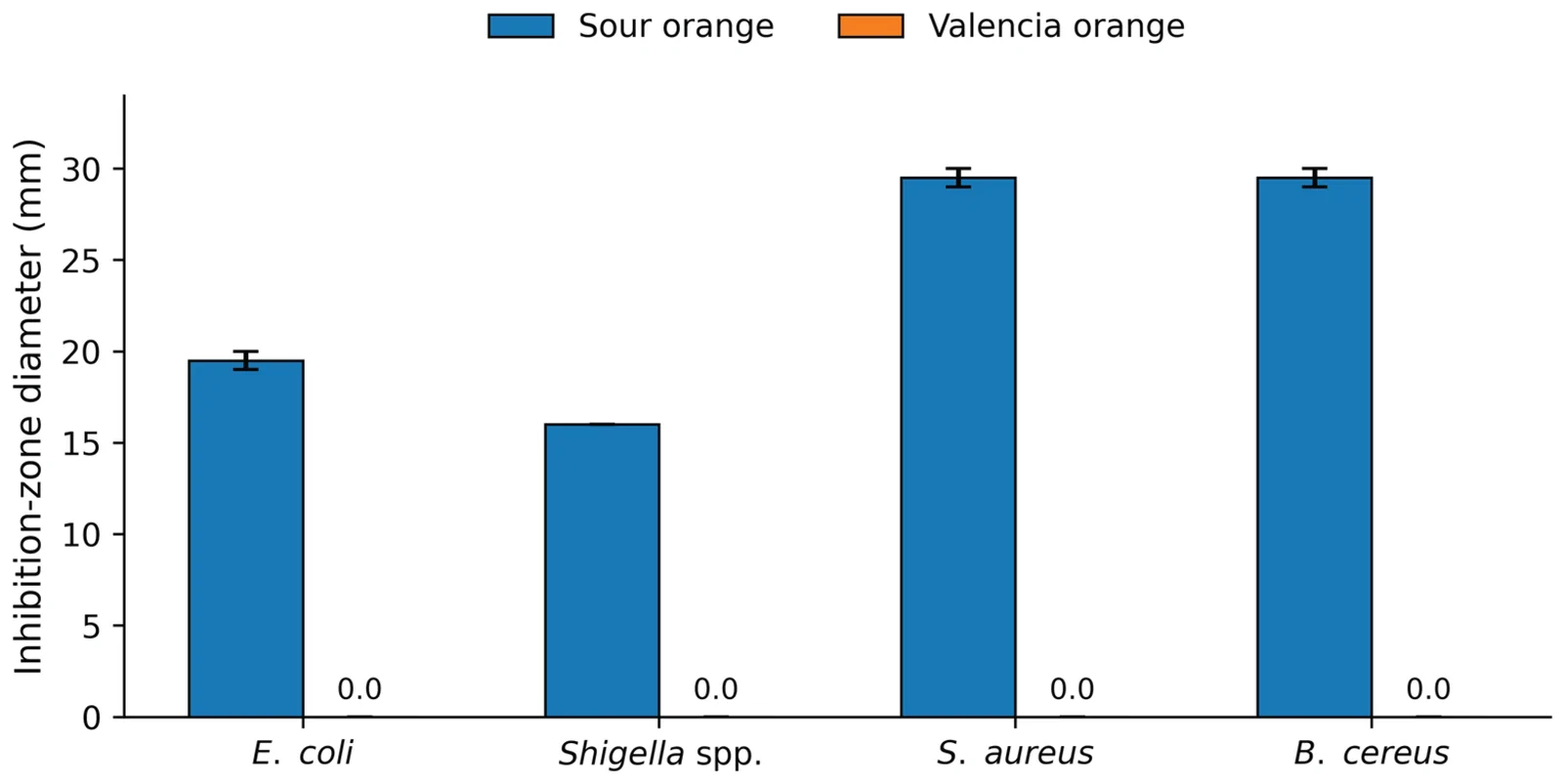
Antimicrobial activity of Valencia orange and sour orange essential oil against four bacterial pathogens. Bars represent the mean ± standard error of two analytical determinations per oil–microorganism condition. The four Valencia orange means are explicitly marked as 0.0 mm at the baseline; no detectable inhibition zone was recorded in either determination. Zone diameters are reported as descriptive screening responses.

**Table 1:** Structure of the analytical determinations used to evaluate antioxidant and antimicrobial activity.

Response Variable	Essential Oil	Assay/Target	Levels	Analytical *n*	Total *N*
Antioxidant capacity (µmol TE/L)	Valencia orange, sour orange	Separate antioxidant assays	ABTS, FRAP	3	12
Inhibition-zone diameter (mm)	Valencia orange, sour orange	Target microorganism	*E. coli*, *Shigella* spp., *S. aureus*, *B. cereus*	2	16

Note. Repeated analytical determinations characterize consistency within each prepared oil batch. The dataset comprises *N* = 12 antioxidant measurements and *N* = 16 antimicrobial measurements; analytical *n* is not treated as independent extraction replication.

**Table 2:** Individual analytical determinations and descriptive antioxidant results.

Variety	Assay	Individual Analytical Determinations (µmol TE/L)	Mean ± SD (µmol TE/L); RSD (%)
Valencia orange	ABTS	431.580; 438.080; 444.582	438.08 ± 6.50; 1.48
Valencia orange	FRAP	200.31; 187.21; 174.10	187.21 ± 13.11; 7.00
Sour orange	ABTS	158.514; 152.012; 158.514	156.35 ± 3.75; 2.40
Sour orange	FRAP	69.27; 69.27; 75.82	71.45 ± 3.78; 5.29

Note. TE = Trolox equivalents. Values are three analytical determinations for each oil–assay condition. Mean ± SD summarizes within-sample analytical consistency. ABTS and FRAP are reported as distinct antioxidant endpoints.

**Table 3:** Individual observations and descriptive inhibition-zone results.

Variety	Microorganism	Individual Observations (mm)	Mean ± SD (mm)
Sour orange	*E. coli*	20; 19	19.5 ± 0.71
Sour orange	*Shigella* spp.	16; 16	16.0 ± 0.00
Sour orange	*S. aureus*	30; 29	29.5 ± 0.71
Sour orange	*B. cereus*	30; 29	29.5 ± 0.71
Valencia orange	*E. coli*	0; 0	0.0 ± 0.00
Valencia orange	*Shigella* spp.	0; 0	0.0 ± 0.00
Valencia orange	*S. aureus*	0; 0	0.0 ± 0.00
Valencia orange	*B. cereus*	0; 0	0.0 ± 0.00

Note. Values are two analytical determinations per oil–microorganism condition. Mean ± SD summarizes within-sample analytical consistency.

**Table 4:** Yield and bioactivity of *C. sinensis* and *C. aurantium* essential oils in the present study and selected previous studies.

Study	Species and Organ	Extraction Method	Yield	Antioxidant Assay (Result)	Antimicrobial Method (Maximum Result)
This study	*C. sinensis*, peel	Steam distillation	1.25 mL/100 g	ABTS 438.08 µmol TE/L; FRAP 187.21 µmol TE/L	Disk diffusion: no detectable inhibition (0 mm) against the 4 bacteria
This study	*C. aurantium*, peel	Steam distillation	1.25 mL/100 g	ABTS 156.35 µmol TE/L; FRAP 71.45 µmol TE/L	Disk diffusion: 29.5 mm against *S. aureus* and *B. cereus* (maximum)
Anwar et al. [10]	*C. sinensis*, peel	Hydrodistillation (50–80 °C)	2.4–3.6% (depending on temperature)	Not evaluated	Diffusion: 14.33 mm against *E. coli*; 11.33 mm against *S. aureus*
Abud Espinoza et al. [39]	*C. sinensis*, seed	Solvent extraction (Soxhlet, 99% ethanol)	17.53%	Not evaluated	Kirby–Bauer: 12 mm against *E. coli*; 14 mm against *S. aureus* (100% oil)
Abud Espinoza et al. [39]	*C. aurantium*, seed	Solvent extraction (Soxhlet, 99% ethanol)	20.80%	Not evaluated	Kirby–Bauer: 15 mm against *E. coli*; 16 mm against *S. aureus* (100% oil)
Ellouze et al. [15]	*C. aurantium*, branch	Hydrodistillation (Clevenger)	0.32%	Not evaluated	Disk diffusion: 25 mm against *E. coli* (50 mg/mL); MIC 1.89 mg/mL

Note. TE = Trolox equivalents; MIC = minimum inhibitory concentration. ABTS and FRAP are distinct chemical assays. Differences in plant organ, extraction procedure, chemical composition, microbial strain, dose, and antimicrobial assay conditions provide useful qualitative context for interpreting the range of values reported across studies.

## Data Availability

The datasets generated and analyzed during the present study are available from the corresponding author upon reasonable request.
